# Effects of Fertilization and Ridge Furrow Planting Patterns on Soil Microbial Communities, Nutrient Dynamics, and Maize Productivity

**DOI:** 10.3390/biology15070551

**Published:** 2026-03-30

**Authors:** Meiling Liu, Zhihui Wang, Ruiqing Zhu, Huichun Xie, Yan Lu

**Affiliations:** 1 Key Laboratory of Tibetan Plateau Land Surface Processes and Ecological Conservation (Ministry of Education), College of Life Sciences, Qinghai Normal University, Xining 810016, China; liuml@lzb.ac.cn (M.L.);; 2 Academy of Plateau Science and Sustainability, Qinghai Normal University, Xining 810016, China; 3 Qilian Mountain Southern Slope Forest Ecosystem Research Station, Haidong 810500, China; 4 Eco-Environmental Research Department, Nanjing Hydraulic Research Institute, Nanjing 210000, China

**Keywords:** fertilization, maize rhizosphere soil, microbial community, sustainable agriculture

## Abstract

This study investigated how fertilization regimes and ridge furrow planting patterns influence the soil nutrient conditions and microbial taxonomic composition and function in the rhizosphere of spring maize in Northeast China. Three treatments were compared: CK (compound fertilizer, small ridge), KF (formula fertilization, small ridge), and BMP (formula fertilization, large double-row ridge). High-throughput sequencing was used to characterize the soil bacterial and fungal community composition and diversity. The results showed that the combination of formula fertilizer and wide-ridge cultivation synergistically improved soil physicochemical properties and significantly increased maize yield (*p* < 0.05). Compared with CK, both BMP and KF significantly improved the composition and diversity of microbial communities. Notably, the BMP treatment increased the relative abundances of Ascomycota and Basidiomycota—key decomposers of soil organic matter, lignin, and cellulose—which suggested enhanced nutrient cycling potential under this integrated management practice. Among the three treatments, BMP (N:P_2_O_5_:K_2_O = 1:2:1, 130 cm wide-ridge double-row planting) achieved the highest maize yield (859 ± 14 kg ha^−1^), representing an 11.0% increase over conventional practices (CK, 774 ± 13 kg ha^−1^). We propose that integrating optimized fertilization with ridge configuration is an effective strategy for improving soil quality, microbial functionality, and crop productivity in Northeast China’s black soil region.

## Simple Summary

Soil degradation from long-term conventional farming in Northeast China’s black soil region threatens maize production and food security, with poor soil health and weakened microbial activity reducing crop yields. This study examined how combining different fertilization methods and ridge planting styles improves soil quality, soil microbial activity, and maize yields by comparing three common farming practices. We found that using tailored fertilizer with wide double-row ridges worked best: this method significantly improved soil nutrient levels, increased maize yields by 11% compared to conventional farming, and boosted the diversity of soil microbes, including key microbes that break down organic matter to cycle nutrients back into the soil. Soil nutrients like nitrogen and organic matter were the main drivers of these healthy microbial communities. This farming approach is a practical, sustainable way to improve both soil health and maize production for local farmers, supporting stable food supplies and long-term agricultural health in this important grain-producing region.

## 1. Introduction

Fertilization is a fundamental agricultural practice that enhances soil nutrient availability, improves soil structure, and increases crop productivity [1]. Beyond its direct effects on plant growth, fertilization plays a critical role in regulating soil microbial activity, improving yield stability, and ensuring food security [2]. Contemporary research demonstrates that fertilization regimes significantly reshape soil microbial communities, which are central to soil biogeochemical cycling and ecosystem functioning [3,4]. Increasing evidence indicates that fertilization regimes can substantially reshape microbial diversity, composition, and functional potential [5], thereby influencing soil fertility and crop performance [6].

Ridge furrow cultivation is widely used to improve water use efficiency and soil conservation, particularly in rain-fed or semi-arid regions. Recent studies suggest that ridge configuration not only modifies soil physical and hydrological properties but also acts as a critical ecological filter, shaping nutrient dynamics and microbial community assembly [7]. However, it remains unclear how this integrated practice specifically regulates critical processes like nitrogen transformation, carbon cycling, and plant–microbe interactions, especially regarding the responses of key microbial functional guilds.

Soil bacteria, the most numerous and widely distributed among soil microorganisms, play a significant role in maintaining soil ecological functions and represent important indicators of soil quality and productivity [8]. Furthermore, soil bacteria facilitate the acquisition of essential nutrients for crop growth through chemical signaling and serve as key drivers of carbon and nitrogen cycling systems [5]. The diversity and abundance of soil bacteria play a significant role in enhancing soil fertility and promoting healthy crop growth [6]. The composition and diversity of soil bacterial communities in agricultural systems are closely linked to the soil microbial environment. Studies utilizing high-throughput sequencing technologies have demonstrated that rhizosphere bacteria can protect crops from pathogens and promote healthy growth by releasing hormones and stress-related signals, thereby enhancing crop resistance [9]. Meanwhile, rational fertilization has been shown to effectively increase the diversity and abundance of soil bacteria in croplands [10], which in turn elevates the overall bacterial biomass and facilitates efficient cycling and transformation of soil carbon and nitrogen [11]. Fertilizers tend to reduce bacterial diversity by 10–25% through pH-mediated selection for copiotrophic taxa such as Proteobacteria over oligotrophic Acidobacteria [3]. Functional outcomes are equally notable: mineral fertilization often stimulates the abundance of *Nitrosomonas* spp. and is associated with 15–30% increases in N_2_O emissions [12].

Among soil microorganisms, in terms of abundance, fungi rank second only to bacteria and play a significant role in the decomposition of soil organic matter and the formation of humus. They are major drivers of soil nutrient cycling and exert important influences on material and energy flows [13]. Fungal communities, which constitute 20–30% of the total soil microbial biomass, are particularly sensitive to agricultural management practices [14]. Ascomycota and Basidiomycota are the dominant phyla involved in decomposition processes, accounting for approximately 70% of the enzymatic activity targeting recalcitrant organic compounds [15]. Within fungal functional groups, some communities exhibit specific ecological functions: Penicillium can degrade various organic compounds such as cellulose, hemicellulose, pectin, lignin, and starch [16]. Mortierella activates soil phosphorus by releasing organic acids, enhances phosphatase activity, provides nitrogen nutrients to plants, and improves disease resistance, thereby promoting plant growth and development [17]. Studies have shown that anthropogenic disturbances significantly affect the structure and diversity of fungal communities in agricultural soils, with soil nutrient status and agriculture management practices being particularly influential factors due to the high sensitivity of soil fungal communities to such changes [18].

Maize (*Zea mays* L.), as China’s largest and most strategically important crop, plays a vital role in food security, animal feed supply, and industrial applications [19]. The Northeast China (NEC) region, forming the core of the nation’s “Golden Maize Belt,” contributes 30–35% of the country’s total maize production and represents one of the nation’s key commercial grain bases [20]. In agricultural production in this area, fertilization, as a crucial means of enhancing nutrient supply, increasing crop yield, and improving crop quality, has attracted widespread attention regarding its impact on soil physicochemical properties. However, decades of continuous monoculture and conventional fertilization practices have led to severe soil degradation. This is reflected in an annual 0.5–1.2% decline in soil organic carbon, a 15–20% reduction in microbial α-diversity indices, and a marked 40% decrease in fungal biomass [21,22]. These changes have substantially compromised critical ecosystem services, particularly the decomposition of recalcitrant organic matter and nutrient cycling efficiency [23]. However, there is limited research on the effect of agriculture management practices on soil microbial characteristics and composition in spring maize in NEC. In particular, the effect of fertilizers and ridge tillage application on the relationship between yield and soil microbes is unknown.

Despite extensive research on fertilization and tillage practices, limited attention has been paid to their combined effects on rhizosphere microbial communities and the associated mechanisms linking soil properties, microbial dynamics, and crop yield. We hypothesize that the integration of fertilization and ridge management may enrich diverse beneficial microbial communities in the soil, thereby increasing soil biodiversity, which is a fundamental driving force of plant productivity and soil function. To address these knowledge gaps, spring maize was cultivated under different fertilization and ridge tillage management practices. We implemented a comprehensive comparison of three management systems: CK (conventional 65 cm single-row with synthetic fertilizer), KF (optimized 65 cm single-row with formula fertilization), and BMP (innovative 130 cm double-row with formula fertilization). To quantify key microbial community parameters, we employed high-throughput sequencing analysis, evaluating α-diversity, β-diversity, and functional guild distributions. The research aims to investigate how these fertilization treatments affect soil microbial ecosystems in spring maize fields, with a focus on revealing their impacts on the community, diversity, and functions of rhizosphere microbes. Our findings provide critical insights for optimizing soil fertilization management within NEC’s intensive maize systems, directly informing national “high-standard farmland” development initiatives.

## 2. Materials and Methods

### 2.1. Study Area

**Figure 1:**
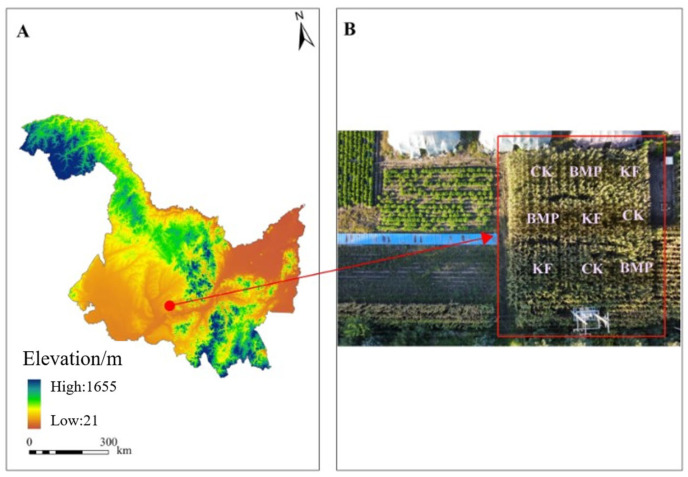
Geographic location of the study area and experimental plot layout. (**A**) Location of Bayan County of Heilongjiang Province in Northeast China. (**B**) Photographic view of the experimental plot showing the three treatment arrangements (CK, KF, BMP) with randomized block design.

The experimental site is located within the Bayan County Agricultural Science Research Institute’s demonstration zone (45°54′28″ N–46°40′18″ N, 126°45′53″ E–127°42′16″ E) in Heilongjiang Province (Figure 1), Northeast China, representing a typical intensive maize production system in the region’s fertile black soil belt. The area experiences a temperate continental monsoon climate characterized by distinct seasonal variability, with a mean annual temperature of 2.9 °C, exhibiting considerable seasonal fluctuations from −25 °C in January to 22 °C in July, accompanied by substantial solar radiation inputs (4800–5200 MJ m^−2^ yr^−1^) and 2640 annual sunshine hours. Precipitation follows a strongly seasonal pattern, with a mean annual total of 560 mm, approximately 70% of which occurs during the summer growing season (June–August), while potential evapotranspiration reaches 850 mm annually. The dominant soil type is classified as Phaeozems, developed from loess parent material with favorable agricultural properties. This experimental site has maintained continuous maize monoculture for over 15 years under conventional management practices, including annual moldboard plowing to 30 cm depth and consistent mineral fertilization inputs at an average of 250 kg N ha^−1^ yr^−1^, 100 kg P_2_O_5_ ha^−1^ yr^−1^, and 120 kg K_2_O ha^−1^ yr^−1^.

### 2.2. Experimental Treatments

In 2019–2023, individual monitoring plots, each measuring 700 cm × 520 cm (approximately 35 m^2^), were established at the demonstration zone of the Bayan County Agricultural Science Research Institute. The soil in the experimental area was classified as heavy loam under the mollisol (black soil) category. Three fertilization and ridge tillage management practices were implemented: CK (control, application of slow-release compound fertilizer (SACF), small ridge planting at 65 cm), KF (formula fertilization, small ridge planting at 65 cm), and BMP (formula fertilization, large ridge double-row planting at 130 cm). The CK treatment involved the application of 45% SACF compound fertilizer (N15–P15–K15%) at a rate of 375 kg ha^−1^ on 65 cm narrow ridges. Both the KF and BMP treatments utilized an identical mineral fertilizer formulation, consisting of 46% urea, 46% triple superphosphate, and 50% potassium sulfate, with an N:P_2_O_5_:K_2_O = 1:2:1, applied at 300 kg ha^−1^. The KF group was maintained, with planting on narrow 65 cm ridges, while the BMP treatment adopted wider 130 cm ridges with a double-row planting pattern. For the CK and KF treatments (65 cm narrow ridges, single-row planting), the row spacing was 65 cm (ridge width), with an intra-row plant spacing of 30.6 cm. For the BMP treatment (130 cm wide ridges, double-row planting), each 130 cm ridge accommodated two rows spaced 50 cm apart, with 30 cm between adjacent ridges (ridge center-to-center = 130 cm). Intra-row plant spacing was maintained at 30.6 cm. All treatments maintained a consistent planting density of 50,250 plants ha^−1^, with fertilizer uniformly applied through manual furrow-opening. Weed control was conducted via manual hoeing and pre-emergence herbicide (Acetochlor) application at ridge formation. Irrigation was managed uniformly across all plots using a sprinkler system, with applications scheduled based on the local conventional practice to maintain adequate soil moisture during the critical growth stages.

### 2.3. Sampling Protocol

The dominant spring maize cultivar of Northeast China was sown in the monitoring plots in May, reaching full maturity by September in each year. After five years of different management practices, periodic surveys were conducted to evaluate ridge-associated agronomic factors. Soil samples were collected during the maize harvest period on 15–20 August 2023. In each plot, five sampling points were selected using an S-shaped sampling pattern to ensure representativeness. Soil cores were collected from the 0–20 cm plow layer using a stainless steel auger (5 cm in diameter). The five cores from the same plot were combined into a single composite sample. Visible roots, stones, and organic debris were removed manually. The fresh soil samples were immediately transported to the laboratory, where they were divided into three subsamples: one was stored at 4 °C for inorganic nitrogen (NO_3_^−^-N and NH_4_^+^-N) analysis; another was flash-frozen in liquid nitrogen and stored at −80 °C for subsequent DNA analysis; and the remainder was air-dried, ground, and sieved (2 mm and 0.25 mm) for the analysis of other physicochemical properties.

### 2.4. Analytical Methods

#### 2.4.1. Determination of Soil Physicochemical Properties

Table 1 shows the methods used for measuring physicochemical properties.

#### 2.4.2. Maize Yield

At maize physiological maturity, characterized by the disappearance of the kernel milk line and the appearance of the black layer, all ears from each treatment plot were harvested. The kernels were subsequently threshed, and their moisture content was determined using a grain moisture meter (Model 8188-A, Shandong Zeshun Electronic Technology Co., Ltd., Dezhou, China). The measured grain yield for each plot was adjusted to a standard moisture content of 14% to obtain the final yield.

#### 2.4.3. Soil Microbial DNA Extraction

Soil microbial genomic DNA was isolated from differentially treated soil samples using a commercial soil DNA extraction kit (MagaBio Soil Genomic DNA Purification Kit (Thermo Fisher Scientific, Shanghai, China)) in strict accordance with the manufacturer’s specifications. Following extraction, DNA concentration and purity were spectrophotometrically quantified to verify quality parameters, with absorbance ratios (A260/280 and A260/230) falling within the optimal range of 1.8–2.0, ensuring suitability for downstream high-throughput sequencing applications.

#### 2.4.4. PCR Amplification and Sequencing

The V3-V4 hypervariable region of the 16S rRNA gene was amplified by PCR using the bacterial-specific primer pair 338F (5′-ACTCCTACGGGAGGCAGCAG-3′) and 806R (5′-GGACTACHVGGGTWTCTAAT-3′). The hypervariable ITS region of fungal ribosomal DNA was amplified using the fungal-specific primer pair ITS1F (forward: 5′-CTTGGTCATTTAGAGGAAGTAA-3′; reverse: 5′-GCTGCGTTCTTCATCGATGC-3′) under optimized PCR conditions.

PCR amplification was conducted in triplicate 20 μL volumes containing 4 μL of 5× buffer, 2 μL of 2.5 mM dNTPs, 0.8 μL of each primer (5 μM), 0.4 μL of polymerase, and 10 ng of template DNA. The thermal cycling conditions were as follows: initial denaturation at 95 °C for 3 min, followed by 27 cycles (for bacteria) or 35 cycles (for fungi) of denaturation at 95 °C for 30 s, annealing at 55 °C for 30 s, and extension at 72 °C for 45 s, with a final extension at 72 °C for 10 min.

Amplification products were subjected to electrophoresis on 1% (*w*/*v*) agarose gels stained with ethidium bromide for visual quality assessment, followed by purification using a QIAquick PCR Purification Kit (QIAGEN, Hilden, Germany). The validated amplicons were subsequently sequenced on the Illumina HiSeq platform (San Diego, CA, USA, 2 × 250 bp paired-end sequencing) to generate high-resolution microbial community profiles, with appropriate negative controls included throughout the experimental procedure to monitor potential contamination.

#### 2.4.5. Data Processing and Analysis

Statistical analyses were conducted using SPSS 22.0 (IBM Corp., Armonk, NY, USA) and figures were generated using R (version 4.0.4). One-way analysis of variance (ANOVA) and Duncan’s multiple comparisons were performed. Linear discriminant analysis (LDA) effect size (LEfSe) was implemented using LEfSe software (http://huttenhower.sph.harvard.edu/galaxy/, accessed on 26 March 2026). Redundancy analysis (RDA) was performed to assess the impact of environmental factors on maize yield and soil microbial community composition using Wekemo Bioincloud (https://www.bioincloud.tech/task-meta, accessed on 26 March 2026).

## 3. Results

### 3.1. Physicochemical Properties and Maize Yield

**Figure 2:**
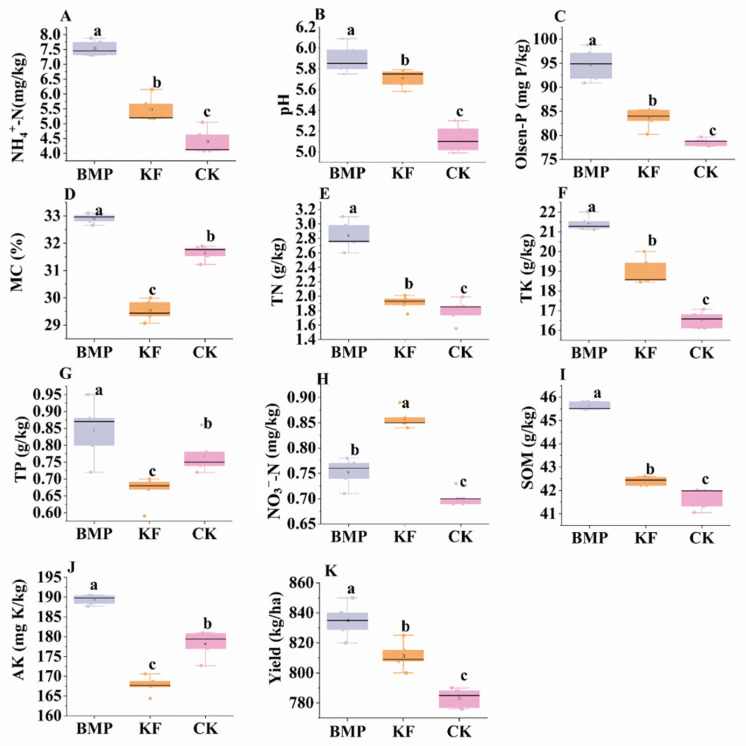
Effects of fertilization and ridge cultivation regimes on soil physicochemical properties and yield in spring maize rhizosphere. (**A**) NH_4_^+^-N; (**B**) pH; (**C**) Olsen-P; (**D**) moisture content; (**E**) TN; (**F**) TK; (**G**) TP; (**H**) NO_3_^−^-N; (**I**) SOM; (**J**) AK; (**K**) maize yield. Note: Different lowercase letters above the box plots indicate significant differences among treatments at *p* < 0.05.

The BMP treatment significantly improved multiple soil physicochemical properties compared with CK. Soil pH increased from 5.1 ± 0.2 to 5.9 ± 0.2 (*p* < 0.05), while NH_4_^+^-N and TN increased by 76% and 61%, respectively (*p* < 0.01). Soil organic matter also showed a moderate increase under BMP (*p* < 0.05). The KF treatment exhibited the lowest TP (0.08± 0.01 g kg^−1^), AK (168 ± 2 g kg^−1^), and moisture content (29.4 ± 0.5%) among the three groups (Figure 2).

Maize yield varied significantly among treatments, with BMP achieving the highest yield (859 ± 14 kg ha^−1^), followed by KF and CK. The yield under BMP was 11.0% higher than that under CK (774 ± 13 kg ha^−1^).

### 3.2. Correlation Analysis of Physicochemical Properties and Maize Yields

**Figure 3:**
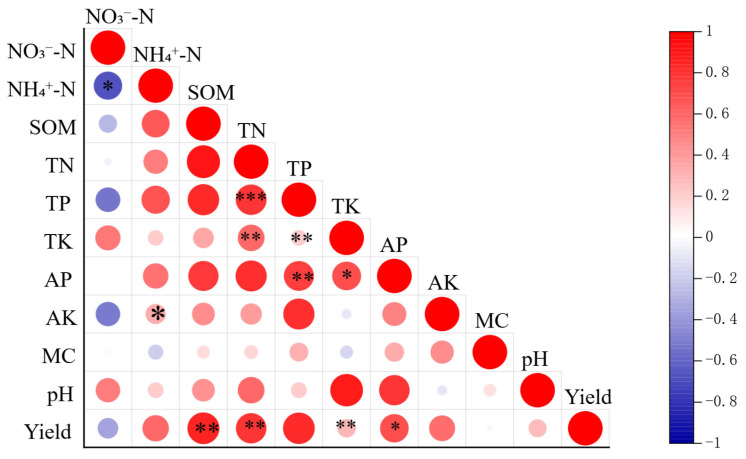
Heatmap of correlations between soil physicochemical properties and yield of spring maize under different fertilization and ridge cultivation regimes. Note: The color depth indicates the strength of positive or negative correlations. Asterisks denote statistical significance: * *p* < 0.05, ** *p* < 0.01, *** *p* < 0.001.

We analyzed the correlation between soil physical and chemical properties and crop yield, revealing significant associations among multiple indicators (Figure 3). At the *p* < 0.01 level, seven groups of highly significant positive correlations were identified: total nitrogen with total phosphorus, total phosphorus with available phosphorus, organic matter with yield, total potassium with yield, total nitrogen with yield, total nitrogen with total potassium, and total phosphorus with total potassium. At the *p* < 0.05 level, four groups of significant correlations were observed. Notably, nitrate nitrogen exhibited the only significant negative correlation with ammonium nitrogen. Conversely, ammonium nitrogen demonstrated significant positive correlations with total potassium, available phosphorus, yield, and both total nitrogen and available phosphorus.

The results of this experiment indicate a strong synergistic relationship among soil organic matter, total nitrogen, total phosphorus, total potassium, and crop yield. Additionally, the content of available phosphorus was significantly influenced by total nutrient (TP, TK, and TN) levels, contributing positively to crop yield. The observed negative correlation between nitrate nitrogen and ammonium nitrogen suggests potential competition or dynamic interactions between these forms of nitrogen during soil transformation.

### 3.3. Diversity of Soil Bacterial Communities

The alpha diversity of the spring maize rhizosphere bacterial communities under different fertilization and ridge cultivation regimes was assessed using the Chao1 and ACE indices (for community richness) and the Simpson and Shannon indices (for community diversity). The results are presented in Table 2. The values for all bacterial indices followed the order BMP > KF > CK. Significant differences were observed between the control (CK) and fertilization treatments (KF and BMP) for both the richness indices (Chao1 and ACE) and the Shannon diversity index. However, no significant difference was found in the Simpson index among the treatments.

The alpha diversity analysis revealed significant variations in fungal community structure among treatments, as quantified by complementary diversity indices (Table 2). The Shannon indices demonstrated significantly higher fungal diversity in the BMP treatment compared to other groups (*p* < 0.05). The Chao1 and ACE showed consistent trends, with both KF and BMP treatments exhibiting significantly elevated values relative to CK, indicating that formulated fertilization regimes substantially enhanced fungal community richness. These results collectively demonstrate that modified fertilization approaches significantly influence both the diversity and richness of rhizosphere fungal communities in spring maize cultivation systems.

**Figure 4:**
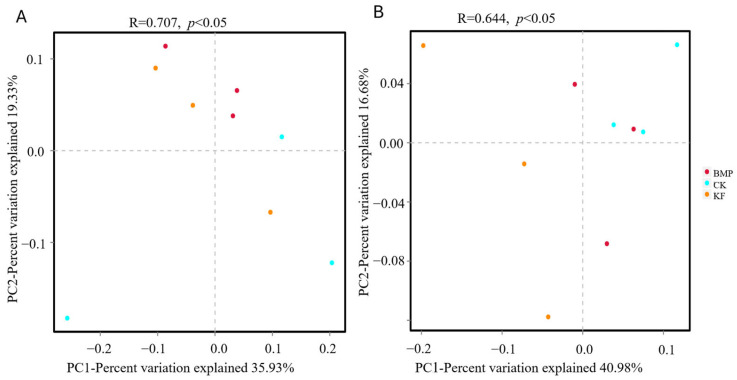
PCoA results of (**A**) bacterial and (**B**) fungal communities found with different fertilization and ridge cultivation regimes. PCoA plot of first two principal components based on operational taxonomic units with different fertilization types.

The PCoA, which is based on Bray–Curtis distances to construct the entire structure of the microbial community, was used to analyze soil microbial β-diversity in the spring maize rhizosphere under different fertilization and ridge cultivation regimes. Bacterial beta diversity indicated that the soil bacterial community composition differed significantly in all treatments (R = 0.707, *p* < 0.05). The main principal components obtained in this study (PC1 and PC2) explained 35.93% and 19.33% of the variance, respectively (Figure 4A). The fungal community composition differed between the three treatments (R = 0.644, *p* < 0.05), with the first two principal components explaining approximately 57.66% of the total variation (PC1: 40.98%; PC2: 16.68%) (Figure 4B). These results implied that the different fertilization types had a key influence on the soil bacterial and fungal community structure and diversity in the spring maize rhizosphere.

### 3.4. Microbial Community Composition and Structure

**Figure 5:**
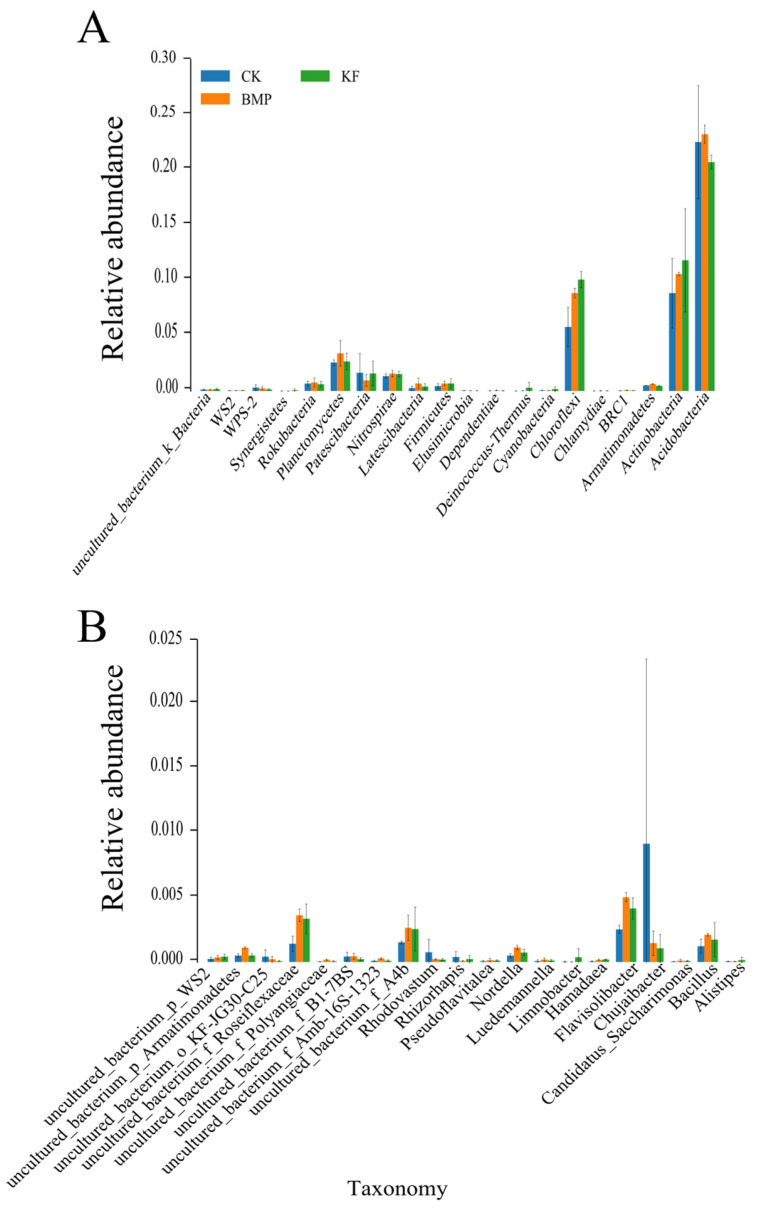
Effects of different fertilization and ridge cultivation regimes on the relative abundance in phylum (**A**) and genera (**B**) of bacterial lineages. The linear discriminant analysis (LDA) effect size (LEFSe) analysis was performed to identify the indicator taxa representing each group, and the values were significant (*p* < 0.05) when the LDA score was over 3.

LEfSe was used to identify the key taxa contributing to the community differences between different fertilization and ridge cultivation regimes on spring maize rhizosphere. The relative abundances of bacterial communities at the phylum level for the three treatment groups are shown in Figure 5A. Acidobacteria and Actinobacteria were the most abundant phyla in all three samples. Acidobacteria showed the highest relative abundance in BMP and remained dominant in KF and CK. Actinobacteria were also consistently abundant, with the highest proportion in KF. Chloroflexi was notably more abundant in sample KF compared to CK and BMP. Several phyla showed low abundances across all samples, including Patescibacteria, Nitrospirae, Planctomycetes, and candidate phyla (Rokubacteria and Synergistete). The relative abundances of specific bacterial genera or uncultured taxa across three samples are shown in Figure 5B. Compared to CK, the Chujaibacteria genus showed a decrease in KF and BMP. Roseiflexaceae, Flavisolibacteria, Nordella, and Bacillus were the dominant genera, showed significant differences in the three treatments, and shared a consistent pattern: BMP > KF > CK.

**Figure 6:**
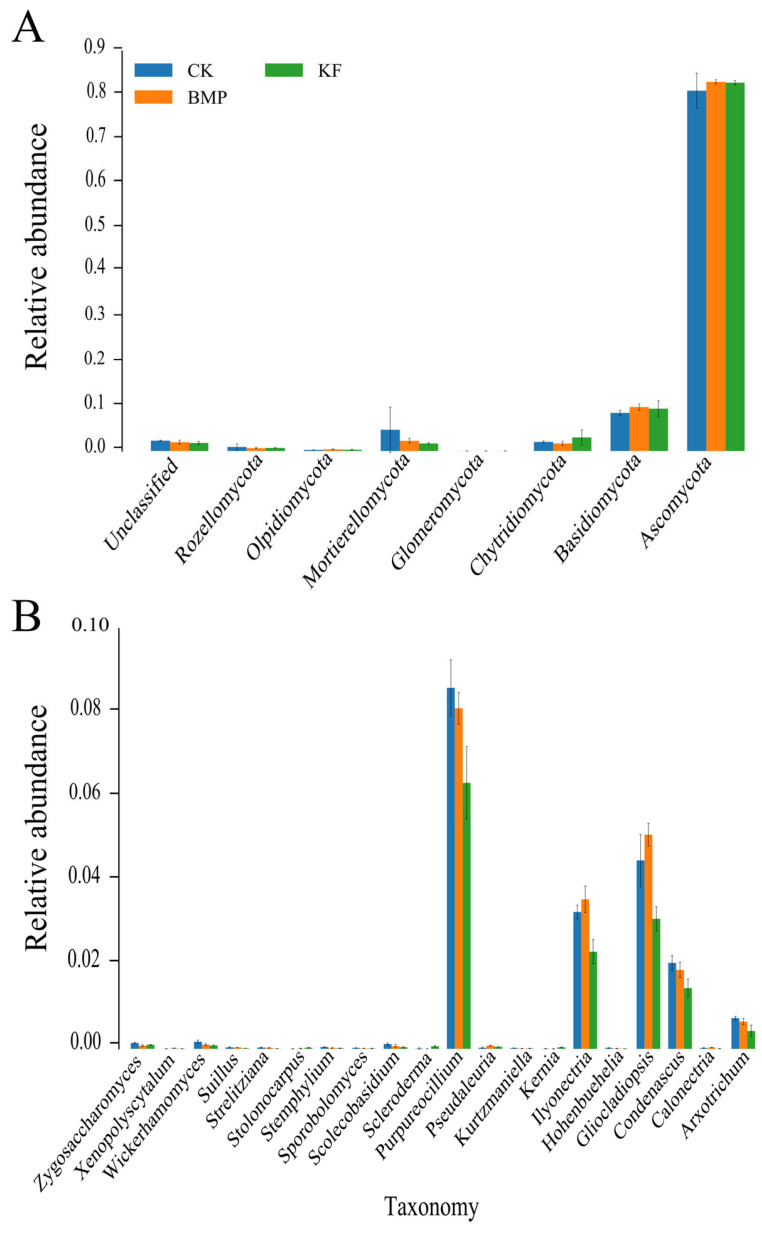
Effects of different fertilization and ridge cultivation regimes on the relative abundance in phylum (**A**) and genera (**B**) of fungal lineages. The linear discriminant analysis (LDA) and linear effect size (LEFSe) analysis were performed to identify the indicator taxa representing each group, and the values were significant (*p* < 0.05) when the LDA score was over 3.

Regarding the soil fungal community composition, the relative abundances of major fungal taxa at the genus level for the three treatment groups are shown in Figure 6. At the phylum level, the dominant fungal taxa across all treatments were Ascomycota, Basidiomycota, Mortierellomycota, Chytridiomycota, Rozellomycota, Olpidiomycota, and Glomeromycota, collectively representing 97.7–98.2% of the total relative abundance. Comparative analysis showed that the relative abundances of Ascomycota, Basidiomycota, and Olpidiomycota followed the order BMP > KF > CK, indicating significant increases compared to CK. In contrast, Chytridiomycota exhibited KF > CK > BMP, with KF showing enrichment relative to CK, while BMP displayed suppression. At the genus level, the dominant fungal taxa across all treatments were Purpureocillium, Gliocladiopsis, Ilyonectria, Condenascus, and Arxotrichum, collectively accounting for 50.1–54.7% of the total relative abundance. Among the fertilized treatments, the relative abundances of Purpureocillium, Condenascus, and Arxotrichum followed the order CK > BMP > KF, all showing decreases compared to CK. Ilyonectria and Gliocladiopsis exhibited BMP > CK > KF, indicating that BMP enhanced their abundances compared to CK, whereas KF led to a decline.

### 3.5. Factors Influencing Soil Bacterial and Fungal Communities

**Figure 7:**
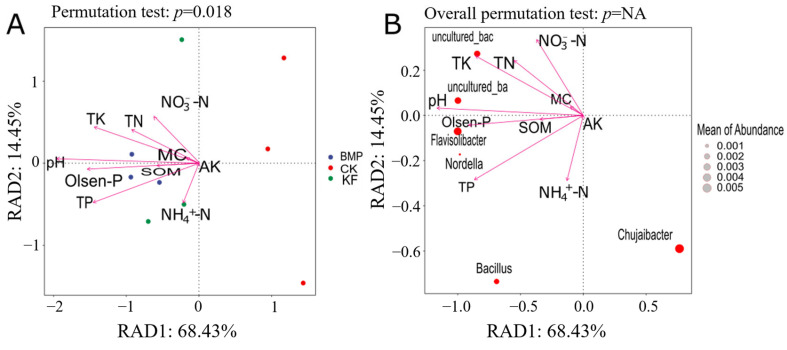
Distance-based redundancy analysis (RDA) of bacterial communities (**A**) and the dominant genus (**B**) with soil physicochemical properties under different fertilization and ridge cultivation regimes.

RDA of bacterial communities with soil physicochemical properties under different fertilization and ridge cultivation regimes was analyzed (Figure 7A). The first two RDA axes explained 82.88% of the variance (RDA1: 68.43%, RDA2: 14.45%; *p* = 0.014). The BMP samples correlated positively with SOM and TN. The KF samples showed a strong association with NH_4_^+^-N. It was shown that NH_4_^+^-N and moisture content were the primary drivers of community variation.

The correlation between the dominant bacterial genus and soil physicochemical properties was also studied (Figure 7B). RDA revealed that soil nutrients (TK, SOM, TN, and Olsen-P) significantly structured microbial communities (RDA1: 68.43%; RDA2: 14.45%). For the dominant bacterial genus, f_Roseiflexaceae showed a negative correlation with total potassium and could be promoted in low-potassium environments. Chujaibacter was positively correlated with organic matter, indicating a preference for environments with high organic matter content. Bacillus was located at the negative quadrant, suggesting potential suppression under high-nitrogen and -phosphorus conditions. Overall, the trends indicate that soil nutrients (particularly TN, Olsen-P, and TK) serve as key drivers shaping the bacterial community structure, with different microbial taxa exhibiting specific responses to soil physicochemical factors.

**Figure 8:**
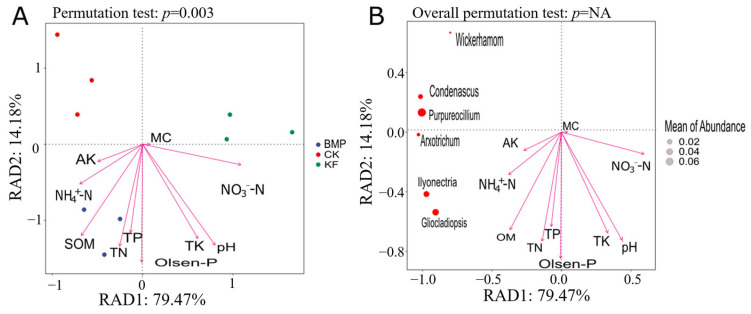
Distance-based redundancy analysis (RDA) of fungal communities (**A**) and the dominant genus (**B**), with soil physicochemical properties under different fertilization and ridge cultivation regimes.

RDA revealed a clear separation among different fertilization and ridge cultivation regimes (Figure 8A). Distance-based RDA showed that the first two components explained 93.65% of the total variation in the fungal community, with the first two axes accounting for 79.47% and 14.18%, respectively. The BMP group clustered in the upper-right quadrant, strongly associated with NH_4_^+^-N, TN, and SOM. In contrast, the KF group correlated with NO_3_^−^-N, while the CK group showed minimal association with soil physicochemical factors.

The correlation between the dominant fungal genus and soil physicochemical properties was also investigated (Figure 8B). RDA revealed that 79.47% of fungal community variation (RDA1) was explained by soil physicochemical properties. Purpureocillium and Gliocladiopsis showed strong positive correlations with ammonium nitrogen, while nitrate nitrogen was negatively associated with most microbial features. SOM and moisture content were linked to Ilyonectria and Condenascus abundance. pH exhibited minimal influence on community structure.

## 4. Discussion

### 4.1. Effect of Fertilization and Ridge Cultivation Regimes on Soil Properties and Maize Yield

Soil nutrient status is crucial for the healthy growth and development of crops. Fertilization plays a pivotal role in spring maize production systems. The application of amended fertilizer can enhance the soil’s nutrient storage capacity, while also helping to optimize soil structure and physicochemical properties, thereby improving fertilizer use efficiency [25]. Ridge geometry not only modifies soil physical and hydrological properties but also acts as a critical ecological filter, shaping nutrient dynamics and microbial community assembly [26]. This study found that the BMP (formula fertilizer combined with ridge furrow cultivation) significantly improved NH_4_^+^-N, NO_3_^−^-N, TN, TP, Olsen-P, and SOM. The underlying mechanisms can be attributed to the synergistic effects of ridge furrow microtopography modification and optimized nutrient supply. Specifically, ridge construction alters surface hydrology by concentrating precipitation into furrows, which enhances deep water infiltration and reduces nutrient runoff losses [27]. Concurrently, the combination with formula fertilizer provides a balanced supply of macro and micronutrients tailored to maize demand, which increases the efficiency of nutrient uptake by roots. This improved uptake efficiency reduces the residual mineral nitrogen (NH_4_^+^-N and NO_3_^−^-N) in the soil that is susceptible to leaching [28].

The maize yield exhibited significant variation in response to different fertilization treatments. Notably, the BMP treatment consistently produced the highest yields in both experimental harvest areas, achieving a mean yield of 859 ± 14 kg ha^−1^. In contrast, the yield of the control (CK) treatment was substantially lower, at 774 ± 13 kg ha^−1^. This marked yield enhancement under BMP aligns with the previously observed improvements in key soil physicochemical properties [29]. The superior yield performance in BMP plots suggests that the formula fertilizer combined with ridge furrow cultivation not only improved soil nutrient status and storage capacity but also translated these soil improvements into enhanced crop productivity. These results support the hypothesis that managing formula fertilizer combined with ridge furrow cultivation like BMP can effectively optimize the soil–plant nutrient continuum, thereby boosting maize yield.

### 4.2. Effect of Fertilization and Ridge Cultivation Regimes on Soil Microbial Communities

Microorganisms play a key role in soil ecosystems, and their quantity and diversity are important indicators for assessing soil fertility [30]. In this study, the results showed that under BMP and KF treatments, the fungal Chao1 and ACE indices (community richness) were higher compared to the control group (CK), while the Simpson index decreased. This indicates that formula fertilization and large ridge cultivation not only increased the species richness of the fungal community but also reduced community evenness. This may be attributed to the BMP treatment improving the soil environment and promoting the proliferation of specific fungal taxa, leading to an increase in the relative abundance of some dominant fungi [20]. The enriched microbial community provides a stable microbial composition for the soil, playing important roles in nutrient cycling, disease prevention, and control [30].

Analysis of the dominant bacterial phyla in the spring maize rhizosphere soil revealed that across all treatments, Acidobacteria and Actinobacteria exhibited the highest relative abundances. This finding is consistent with previous studies [31], indicating that dominant phyla occupy broad ecological niches and can adapt to various environmental conditions, albeit with varying proportions. Our inter-group analysis found that the relative abundance of Actinobacteria across treatments followed the order KF > BMP> CK, while that of Acidobacteria followed BMP > CK > KF. The relative abundance of Acidobacteria is indicative of soil nutrient status, showing a negative correlation with soil quality; lower abundance is associated with higher soil quality [3]. In contrast, Actinobacteria are predominantly saprophytic, degrading complex lignin and cellulose to enhance soil nutrient content and facilitate organic matter assimilation and turnover [32]. This suggests that the dominant bacterial phyla in the KF treatment may be more effectively adapted to the cultivation conditions. At the genus level, the observed abundance pattern (BMP > KF > CK) for Bacillus and Flavisolibacter provides in situ evidence supporting their proposed roles in soil fertility enhancement. The dominance of Bacillus under BMP aligns with its known metabolic capabilities, namely, the solubilization of recalcitrant P and K and N_2_ fixation in diazotrophic strains [33], which likely contributed to the increased nutrient availability in this treatment. Furthermore, the enrichment of Flavisolibacter under the same treatment corroborates its classification as a fertilization-responsive beneficial genus [34]. This result indicates that BMP can increase the abundance of beneficial bacteria. Ascomycota emerged as the dominant phylum for fungi, consistent with previous findings [35,36], followed by Basidiomycota, Mortierellomycota, and Chytridiomycota. As the primary agents of organic matter decomposition, Ascomycota [37], together with Basidiomycota, the key lignocellulose degraders [38], constitute the core fungal workforce driving soil carbon cycling. BMP treatment showed the highest relative abundances of both Ascomycota and Basidiomycota (BMP > KF > CK), suggesting that BMP is better adapted to the specific conditions of the planting environment. The ridge furrow cultivation combined with formula fertilization optimizes the soil microclimate and nutrient availability, thereby stimulating fungal proliferation and activity.

### 4.3. Relationship Between Soil Microbial Communities and Soil Physicochemical Properties

Most previous research has indicated that soil pH, SOM, and MBC are critical factors that shape variations in the soil microbial community. Fertilizers may be absorbed and used by plants or remain in the soil after application [29], resulting in altered protists, bacteria, and fungal communities [6,39]. The positive correlation of BMP samples with SOM and TN suggests that formula fertilization associated with large ridge tillage enhances conditions for a copiotrophic bacterial community, favoring taxa that thrive in resource-rich environments. In contrast, the strong association of KF samples with NH_4_^+^-N highlights a unique nitrogen dynamics regime, potentially driven by formula fertilization strategies [40]. Crucially, the identification of NH_4_^+^-N and moisture content as primary drivers of overall community variation aligns with established ecological principles, as both factors are critical for microbial metabolism, osmotic balance, and the diffusion of substrates.

Delving into the responses of individual dominant bacterial genera reveals the mechanistic link between soil nutrients and community assembly. The significant structuring of communities by key soil nutrients (TK, SOM, TN, and Olsen-P) is reflected in the specific niche adaptations of different bacterial taxa. The negative correlation between f_Roseiflexaceae and total potassium is particularly noteworthy. Members of this family, often associated with phototrophic or anoxygenic phototrophic lifestyles in some environments, appear to possess a competitive advantage in low-potassium conditions [41]. Conversely, the positive correlation of Chujaibacter with organic matter exemplifies a classic copiotrophic strategy. This genus likely harbors the enzymatic machinery to efficiently decompose complex organic compounds, making it a successful competitor and key player in carbon cycling within high-SOM environments [42]. The case of Bacillus, positioned in the negative quadrant relative to the nitrogen and phosphorus vectors, suggests suppression under high TN and Olsen-P conditions. While many Bacillus species are known for their metabolic versatility and stress tolerance, they may be outcompeted in such nutrient-saturated environments by faster-growing strategists or be sensitive to specific biochemical changes that often accompany heavy nitrogen and phosphorus fertilization [43].

Regarding fungal communities, BMP significantly increased the relative abundances of Ascomycota and Basidiomycota, key decomposer phyla, while simultaneously reducing certain beneficial genera such as Penicillium and Mortierella compared to CK. Our RDA results revealed that BMP samples were strongly associated with NH_4_^+^-N, while KF samples correlated with NO_3_^−^-N. This shift toward ammonium dominance under BMP has profound implications for fungal community assembly. Ascomycota and Basidiomycota, particularly their saprotrophic members, generally exhibit preferential assimilation of reduced nitrogen forms (NH_4_^+^) over oxidized forms (NO_3_^−^), as ammonium requires less energy for incorporation into amino acids [33,34]. The positive correlation of Purpureocillium and Gliocladiopsis specifically with NH_4_^+^-N supports this interpretation. Both genera include species that are efficient ammonium utilizers and competitive saprobes in organic-rich environments [44,45]. Conversely, the reduction in Penicillium and Mortierella under BMP may reflect competitive displacement rather than direct inhibition. In ammonium-rich environments with elevated SOM, fast-growing decomposers (certain Ascomycota) may outcompete these genera for colonization sites on organic substrates [33]. Additionally, Penicillium species are known to prefer slightly acidic conditions [34], while BMP increased soil pH, potentially favoring other taxa. The BMP-induced shift toward ammonium-dominated nitrogen cycling, coupled with enhanced decomposer activity, suggests a more closed, conservative nutrient cycling system. The increased abundance of lignin-decomposing Basidiomycota indicates enhanced potential for recalcitrant carbon turnover, which may contribute to long-term soil organic matter dynamics [46]. These functional shifts, mediated by both nutrient form and physical habitat modification, position BMP as a strategy that not only improves immediate crop productivity but also enhances underlying soil ecosystem processes.

## 5. Conclusions

This study demonstrates that integrating formula fertilization with optimized ridge furrow planting significantly enhances soil quality, microbial community structure, and maize productivity. The BMP strategy promotes a microbial community dominated by key decomposers, indicating improved nutrient cycling capacity. These findings highlight the importance of coordinated soil management practices in sustaining agricultural productivity and soil ecosystem functions in Northeast China’s black soil region.

## Figures and Tables

**Table 1:** Methods for determining physicochemical properties.

Indicator	Method	Reference
Soil moisture content	Gravimetric method	[24]
pH	Potentiometric method (water/soil ratio of 2.5:1)	[24]
Soil organic matter (SOM)	Potassium dichromate–concentrated sulfuric acid external heating method	[24]
Total nitrogen (TN)	Combustion method	[24]
Total phosphorus (TP)	Sodium hydroxide fusion and molybdenum antimony colorimetric method	[24]
Total potassium (TK) and available potassium (AK)	Flame photometry	[24]
Nitrate nitrogen (NO_3_^−^-N) and ammonium nitrogen (NH_4_^+^-N)	Ultraviolet spectrophotometry and indophenol blue colorimetry	[24]
Available phosphorus (Olsen-P)	Sodium bicarbonate leaching and molybdenum antimony colorimetric method	[24]

**Table 2:** Effects of fertilization on α-diversity of microbial communities in rhizosphere soil of spring maize.

Categories	Treatment	Simpson	Chao1	ACE	Shannon
Bacterial	CK	0.98 ± 0.01 a	1268.97 ± 23.15 b	1271.31 ± 2.80 c	7.67 ± 0.06 ab
	KF	0.97 ± 0.08 a	1310.73 ± 13.54 a	1282.40 ± 1.99 b	8.40 ± 0.55 b
	BMP	0.94 ± 0.02 b	1323.24 ± 10.63 ab	1296.39 ± 4.41 a	8.49 ± 0.07 a
Fungal	CK	0.97 ± 0.02 a	1228 ± 15.89 b	1194 ± 32.04 b	6.22 ± 0.21 b
	KF	0.85 ± 0.02 ab	1260 ± 17.46 b	1244 ± 42.95 ab	6.34 ± 0.06 ab
	BMP	0.79 ± 0.08 b	1302 ± 14.17 a	1290 ± 21.66 a	6.61 ± 0.21 a

Note: Data in the table are presented as mean ± standard deviation. Different letters indicate statistically significant differences at *p* < 0.05.

## Data Availability

The original contributions presented in this study are included in the article. Further inquiries can be directed to the corresponding author.

## Abbreviations

The following abbreviations are used in this manuscript:

CK	compound fertilizer, small ridge
KF	formula fertilization, small ridge
BMP	formula fertilization, large double-row ridge
